# The Impact of Responsive Feeding Practice Training on Teacher Feeding Behaviors in Tribal Early Care and Education: The Food Resource Equity and Sustainability for Health (FRESH) Study

**DOI:** 10.1093/cdn/nzz105

**Published:** 2019-09-20

**Authors:** Kaysha Sleet, Susan B Sisson, Dipti A Dev, Charlotte Love, Mary B Williams, Leah A Hoffman, Valarie Blue Bird Jernigan

**Affiliations:** 1 Department of Nutritional Sciences, University of Oklahoma Health Sciences Center, Oklahoma City, OK, USA; 2 Department of Child, Youth and Family Studies, College of Education and Human Sciences, University of Nebraska Lincoln, Lincoln, NE, USA; 3 Center for Indigenous Health Research and Policy, Oklahoma State University, Tulsa, OK, USA; 4 Department of Biostatistics and Epidemiology, College of Public Health—Schusterman Center, University of Oklahoma Health Sciences Center, Tulsa, OK, USA

**Keywords:** Native American, provider, healthy feeding, teacher, preschool, child care, community-based participatory research

## Abstract

**Background:**

Establishing healthy eating habits early affects lifelong dietary intake, which has implications for many health outcomes. With children spending time in early care and education (ECE) programs, teachers establish the daytime meal environment through their feeding practices.

**Objective:**

We aimed to determine the effect of a teacher-focused intervention to increase responsive feeding practices in 2 interventions, 1 focused exclusively on the teacher's feeding practices and the other focused on both the teacher's feeding practices and a nutrition classroom curriculum, in ECE teachers in a Native American (NA) community in Oklahoma.

**Methods:**

Nine tribally affiliated ECE programs were randomly assigned to 1 of 2 interventions: *1*) a 1.5-h teacher-focused responsive feeding practice training (TEACHER; *n* = 4) and *2*) TEACHER plus an additional 3-h training to implement a 15-wk classroom nutrition curriculum (TEACHER + CLASS; *n* = 5). Feeding practice observations were conducted during lunch at 1 table in 1 classroom for 2- to 5-y-olds at each program before and 1 mo after the intervention. The Mealtime Observation in Child Care (MOCC) organizes teacher behaviors into 8 subsections. Descriptive statistics and the Shapiro–Wilk test for normality were calculated. Paired *t* tests were calculated to determine change in each group.

**Results:**

A mean ± SD of 5.2 ± 2.0 (total *n* = 47) children and 1.7 ± 0.5 (total *n* = 14) teachers/center were observed at baseline, and 5.6 ± 1.7 (total *n* = 50) children and 1.7 ± 0.7 teachers (total *n* = 14) were observed/center postintervention. Total MOCC scores (max possible = 10) improved for TEACHER (6.1 ± 0.9 compared with 7.5 ± 0.3, *t* = 4.12, *P* = 0.026) but not for TEACHER + CLASS (6.5 ± 0.8 compared with 6.4 ± 1.0, *t* = −0.11, *P* = 0.915). No other changes were observed.

**Conclusions:**

Teacher intervention–only programs demonstrated improvements in responsive feeding practices, whereas the programs receiving teacher and classroom training did not. Greater burden likely decreased capacity to make changes in multiple domains. We demonstrated the ability to implement interventions in NA ECE. Further research with larger communities is necessary. This trial was registered at clinicaltrials.gov as NCT03251950.

## Introduction

Cardiovascular disease and cancer are the leading causes of death in the United States, resulting in 633,842 and 595,930 deaths in 2015, respectively (1). Obesity is strongly associated with both cardiovascular disease and some cancers in adulthood (2–5). Children who are overweight or obese in early childhood (ages 2–5 y) have a higher likelihood of remaining obese as adolescents and adults (6). For this reason, the National Academy of Medicine recommends that interventions for obesity prevention to reduce lifetime disease risk begin before the age of 5 y (7).

Thirty-eight percent of children who attended a tribally affiliated early care and education (ECE) program in Oklahoma were overweight or obese in 2011 (8); this rate is higher than the national mean of 21% that same year (9). Cross-sectional, retrospective, and longitudinal cohort design studies provide observational data that ECE experiences influence a child's weight status (10). Observational classroom studies demonstrate that teachers help shape a child's food intake and eating behaviors through feeding practices implemented in the classroom (11, 12). Another observational study working with Native American (NA) ECE programs in Oklahoma reported that teacher feeding practices were one of the most influential components of the nutrition environments affecting children's dietary intake (13). Albeit not in the ECE setting, randomized controlled trials have demonstrated improvements in parental feeding practices and children's nutrition outcomes (14, 15).

Feeding practices are behaviors that teachers use to influence children's dietary intake and are categorized as responsive feeding practices or controlling feeding practices (16). Responsive feeding practices have been shown to support children's acceptance of new foods and ability to self-regulate energy intake (17–20). The Academy of Nutrition and Dietetics has identified 7 key responsive feeding practices (21). One study conducted in Oklahoma ECE programs, half of which were tribally affiliated, found that asking children about their hunger and fullness before and during a meal increased the amount of fruit children tasted and decreased the amount of high-fat/high-sugar foods and fried meats tasted (22). Teachers’ enthusiastic role modeling and talking with children about healthy foods have been associated with healthier eating habits (11, 23, 24). ECE teachers can help reduce lifetime disease burden by instilling positive eating behaviors related to self-regulation of food intake of preschool-aged children in their care (25). Despite the benefits of healthful feeding practices, many teachers use controlling feeding practices which include pressuring children to eat healthy foods, praising children for finishing all of their food, and offering energy-dense foods as rewards, in misguided attempts to promote healthy eating (26, 27). Controlling feeding practices are associated with undesirable outcomes such as consumption of energy-dense foods, lack of self-regulation, and fussy or emotional eating behaviors (28).

Most teachers have not been trained on nutrition and feeding practices but want children to have the best care and to be healthy (29). They have expressed the need to learn strategies to encourage children to try new foods such as fruits and vegetables, manage children's food refusal, and have the desire to promote health in their classrooms (26, 29). Targeted education may improve teachers’ feeding practices and have a positive impact on children's nutrition (26, 30). Also, when teachers are knowledgeable that children can self-regulate their energy intakes, they are more likely to use responsive feeding practices (31). However, although previous studies have surveyed teachers about feeding practice training opportunities and their perceived effects (26, 27), the impact of training on these perceptions and practices has not been evaluated. It is recommended that the content and level of feeding practice training required by ECE teachers to ensure healthful feeding practices are evaluated (27). Although studies in families show impact (14, 15), teachers’ feeding practices training and its effect on teachers’ feeding behaviors have not been thoroughly examined (26), particularly in rural tribally affiliated ECE programs. Given the disproportionate prevalence of chronic disease in NA populations and the importance for early disease prevention through the development of healthy lifestyle behaviors, greater understanding of intervention effectiveness on teacher feeding practices is warranted. Therefore, the purpose of this community-based participatory research (CBPR) study was to compare the effect of *1*) a teacher-focused intervention to increase responsive feeding practices and *2*) the combination of an intervention focused on the teacher's feeding practices and a nutrition classroom curriculum in ECE teachers in an NA community in Oklahoma. We hypothesized that both interventions would improve responsive feeding practices and there would be no difference between the 2 intervention arms.

## Methods

### Study design

This brief randomized intervention study compared teacher feeding practices over lunch in 9 tribally affiliated ECE program classrooms in Osage Nation. All programs were assigned to 1 of the 2 interventions. Four programs participated in a 1.5-h teacher-focused responsive feeding practice training (TEACHER). Five programs both participated in the responsive feeding practice training (1.5 h) and also received a 3-h training to implement a 15-wk classroom nutrition curriculum (TEACHER + CLASS). The 2 trainings were held within 2 wk of each other. At each program, baseline and 1-mo postintervention classroom observations were conducted in the same single classroom with children aged 2–5 y. This study was reviewed and approved by the University of Oklahoma Health Sciences Center Institutional Review Board and the Osage Nation government, which serves as the governing body for any research conducted within Osage Nation.

### Community, executive committee, and participants

A CBPR study known as Food Resource Equity and Sustainability for Health (FRESH) within the Osage Nation tribal community enrolled 9 ECE programs across 4 communities (Skiatook, Fairfax, Hominy, and Pawhuska). An executive committee comprised of community and university partners from several divisions and disciplines guided the entire study from conception to completion. Osage Nation operates 4 Head Start programs, 1 in each of the 4 aforementioned towns, and 4 WahZahZhi Early Learning Academies programs that serve Native families with children aged 2–5 y, 1 in each of the same 4 towns. In addition, Osage Nation operates a Language Immersion School in Pawhuska that serves children of ages 2–5 y as well as other age groups. All 9 ECE programs agreed to participate and the 4 communities with all ECE sites were randomly assigned to either the teacher-focused responsive feeding practice training (TEACHER; *n* = 4) or the responsive feeding practice training plus training on the 15-wk classroom nutrition curriculum (TEACHER + CLASS; *n* = 5).

### Teacher training on healthy feeding practices

While conducting interviews as part of the CBPR process, the Osage Nation executive committee expressed their desire to create a holistic approach to exposing children to fruits and vegetables including the way teachers communicate with children during mealtimes. The Academy of Nutrition and Dietetics best practice feeding behaviors (21) were introduced to a group of stakeholders such as teachers, cooks, and program directors. These stakeholders decided which behaviors they felt were pertinent to include in the training. The research team responded to these identified needs with a teacher-focused training that lasted ∼1.5 h. The training utilized components of the Ecological Approach To Family Style Dining Intervention (EAT Family Style Intervention) including role modeling, peer modeling, sensory exploration, supporting self-regulation, supporting children serving themselves, and rewards and praise (32). Topics selected from the EAT Family Style intervention were guided by the CBPR process and did not include cultural adaptations, per se, but were tailored to the needs expressed by this community. The EAT Family Style intervention was developed to demonstrate these recommended feeding practices through videos and actionable strategies within the natural child care classroom setting and also included strategies to overcome teachers’ barriers for implementing responsive feeding (33, 34). Each discussion topic included handouts that included key messages and verbal prompts regarding responsive feeding for teachers to refer to. Video examples of teachers in classrooms with 2- to 5-y-old children accompanied each topic (34). Small- and large-group discussions were utilized during the role modeling and supporting self-regulation topics to allow teachers to demonstrate understanding and provide practice scenarios. An outline of the training is listed in **Table 1**.

**TABLE 1:** Description of teacher-focused responsive feeding practice teacher training received by all 9 programs including activities and materials provided

Section	Section content description	Activities	Material provided
Introduction/background/philosophy (15 min)	• Introduction of speaker• Philosophy on provider feeding behaviors• Introduction activity “What will you say to John”	• Small-group activity (4 min)—response to child's mealtime behavior (refusing to try, eating all food on plate, etc.)• Large-group activity (4 min)—discuss providers’ reactions to child's mealtime behavior	• Activity worksheet
Presentation of research findings (6 min)	• Explain that science has found controlling feeding practices (pressure, restriction, rewards, and preselected portions) to be counterproductive in improving a child's mealtime behavior		
Role modeling (23 min)	• Explain role modeling and use of responsive language• Provide examples of responsive language• Tips for role modeling	• Video (4 min)—role modeling• Video (2 min)—disliking foods• Small-group activity (4 min)—discuss what providers understood, changes they could make, and questions they had• Large-group activity (8 min)—discuss providers’ specific questions and concerns about children's mealtime behaviors	• Handout—Strategies to Model Healthy Eating at Mealtime• Handout—Be a Healthy Role Model for Children
Peer modeling (10 min)	• Explain how to encourage peer modeling• Provide examples of responsive language to utilize peer models	• Video (5 min)—strategies for managing food refusal	• Handout—Peer Modeling Planning Steps for Mealtime• Handout—Healthful Tips for Picky Eaters
Sensory exploration (2 min)	• Introduce what sensory exploration is		• Handout—Food-based Sensory Exploration
Support self-regulation (12 min)	• Explanation of self-regulation• Dispel misunderstandings about children's ability to self-regulate	• Video (3 min)—supporting children's self-regulation• Large-group activity (1 min)—discuss what providers understood, changes they could make, and questions they had	• Handout—Strategies for Supporting Children's Self-Regulation in Eating
Children serve themselves (6 min)	• Identify different skills needed for children to serve themselves• Identify strategies to develop those skills		• Handout—Teaching Children Self-Serving Skills during Play
Praise and rewards (7 min)	• Discuss appropriate use of rewards and praise• Provide examples of responsive language		• Handout—Using Praise Effectively
Closing thoughts (8 min)	• Repeat introduction activity “What will you say to John”• Discuss changes in providers’ responses• Closing thoughts	• Small-group activity (2 min)—response to child's mealtime behavior (refusing to try, eating all food on plate, etc.)• Large-group activity (3 min)—discuss providers’ reactions to child's mealtime behavior	• Activity worksheet

### Classroom nutrition curriculum

Teachers in communities assigned to the TEACHER + CLASS intervention participated in 3 h of training for the classroom curriculum. The classroom nutrition component was a 15-wk curriculum designed to take ∼3 h/wk with the goal of increasing intake of fruit and vegetables. The curriculum provided repeated exposures to 6 target vegetables: tomatoes, bell peppers, spinach, butter beans, squash, and carrots. Curriculum activities were designed to be implemented across 3 d/wk. However, teachers were given flexibility to administer the curriculum however they wanted, according to other curriculum scheduling considerations. There were 3 main curriculum components each week, including an introductory activity, such as a book or song; a sensory activity that allowed all children to explore the vegetable of the week with all 5 senses, including an opportunity to taste the vegetable; and a cooking activity in which the children assisted the teacher in preparing a simple recipe. Children were then provided with a “Take-Home Kit” that allowed the children to prepare the recipe again in the home setting with parents and/or caregivers. Teachers were asked to complete weekly process evaluations giving feedback on the curriculum. Development of the classroom curriculum, intervention fidelity, and outcomes will be described in subsequent articles currently in preparation.

### Measures

#### Demographic information

Program managers completed a demographic questionnaire including education requirements of teachers and nutrition policies. Demographic characteristics of the teachers were not collected. It is noteworthy that, although these programs were operated by Osage Nation, it is likely that not all staff and teachers would identify as NA.

#### Mealtime Observation in Child Care

The Mealtime Observation in Child Care (MOCC) is an observation tool designed to measure the teachers’ responsive feeding practices during mealtime based on previously validated tools (35–37) and the best practice feeding domains identified by the Academy of Nutrition and Dietetics (21). The MOCC tool contains 71 questions divided into 8 sections congruent with the identified best practices (21).

One classroom consisting of 2- to 5-y-old children was selected by the program director and observed at each of the 9 sites twice, once before the training and once ∼1 mo after the teacher training. Interrater reliability for the MOCC is >0.8. However, 1 trained researcher, who was not involved in delivering the teacher training, conducted all observations to minimize interrater reliability concerns. Observer training included classroom experiences to learn about feeding practices, the tool, and the protocol. The observer was instructed to look for verbal and nonverbal teacher interactions with the children and trained on how to score teachers’ statements and actions. Practice observations in a field setting were also completed. Discussion, clarification, and debriefing occurred with the research team after practice observations. Protocol and tool concerns discovered during practice observations were discussed with the MOCC co-developers until modifications were agreed upon.

The trained observer arrived at the identified classroom ∼15 min before lunch. During this time, the observer would record the classroom environment and menu items being served. Meal start time was recorded when the first child at the identified table began eating. The end time was recorded when the last child at the identified table stopped eating. The observer recorded the interactions between all teachers and children sitting at the identified table. If the teachers had to leave the table during the meal and no teachers were left sitting at the table, interactions were recorded when teachers or cooks came near the table and interacted with the children. If the teachers switched tables, the researcher would continue to record the interactions happening at the originally identified table. Throughout lunch, the trained observer would watch for teacher cues, mealtime feeding practices, and responsive language used and document those on the MOCC tool.

Sixty-five of the 71 questions provided the opportunity for the researcher to observe the teacher and respond to their use of the recommended feeding practice as “no, not observed,” “yes sometimes (1–2 times),” “yes regularly ≥3,” or “unable to observe or not applicable” for each behavior. A behavior was coded as unable to observe when it was not applicable to be observed within the mealtime context. For example, if no vegetable or fruit was served, then the item asking if the teacher ate vegetables was coded as “unable to observe.” However, if vegetables were served and the teacher was not eating vegetables then the response was “no, not observed.”

Responses were converted to a numerical scale and summed for each section. Responses were assigned 0 for the less favorable option and 1 for the more favorable option. Any questions marked as “unable to observe” were deducted from the total possible points scored, and thus did not affect the score. Total points were summed for each section and divided by the total possible points for that section. The mean for each section was then multiplied by 10, resulting in a maximum score of 10 for each section. Therefore, the equation for each section is the sum of the section's total points earned divided by the sum of the section's total possible points (subtracting questions scored n/a from the total possible) and multiplied by 10. The total score was scored in the same way as each section, by averaging all of the sections’ scores adjusting for any sections that were unable to be scored, thus it too has a maximum possible score of 10.

### Data analysis

Descriptive statistics, including means, SDs, and frequencies, were calculated for ECE program demographics and MOCC scores. A Shapiro–Wilk test was used to determine normality of the MOCC section and total scores at both time points for both groups. Paired *t* tests were employed to examine differences between baseline and postintervention scores for each of the 8 sections and total scores for all programs within each group (TEACHER and TEACHER + CLASS). Visual observation of raw data scores was used in sections with a small sample size and limited variability to assess change. Because this was a CBPR study conducted in collaboration with Osage Nation and all of the tribe's ECE programs participated, we did not calculate a power analysis. SPSS version 22 (IBM) was used for data analysis.

## Results

### Descriptive statistics

Among the 9 sites, 5 reported the facility had been operating for ≥10 y, 2 reported operating for 3 y, and 2 reported operating for 2 y. Seven sites reported including written policies about nutrition training and professional development for staff, whereas 5 reported including written policies for children's nutrition lessons, and 4 reported including written policies for parent nutrition lessons. See **Table 2** for program descriptive characteristics. Each program serves a mean number of 45.2 ± 22.8 children, ranging between 16 and 95 children. The mean numbers of children and teachers sitting at each observed table at baseline were 5.2 ± 2.0 and 1.7 ± 0.5, respectively. At postintervention the mean numbers of children and teachers sitting at each observed table were 5.6 ± 1.7 and 1.7 ± 0.7, respectively. Although unique identifiers were not collected for children or teachers, 47 children and 15 teachers were observed at baseline and 50 children and 14 teachers were observed postintervention.

**TABLE 2:** Descriptive characteristics of 9 early care and education programs in Osage Nation

Variable	Frequency	%
Years in operation		
2	2	22.2
3	2	22.2
≥10	5	55.6
Minimum provider education requirements		
High school	7	77.8
4-y college graduate	2	22.2
Continued education requirement		
Yes	9	100
Written nutrition education policies		
Staff training	7	77.8
Education for children	5	55.6
Education for parents	4	44.4

**FIGURE 1:**
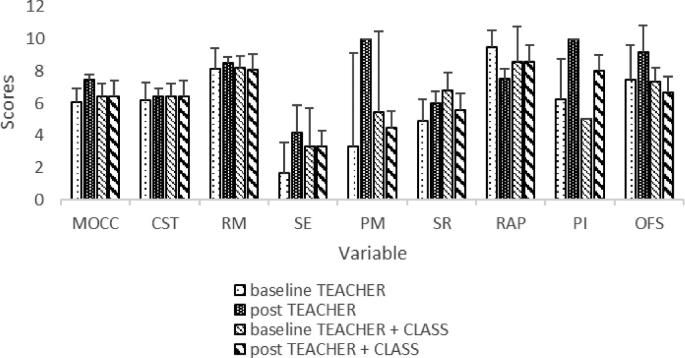
Baseline and postintervention MOCC total and section scores. Bars without SD whiskers had no variation. CST, Children Serve Themselves; MOCC, Mealtime Observation in Child Care; OFS, Overall Feeding Style; PI, Permissiveness/Indulgence; PM, Peer Modeling; RAP, Rewards and Praise; RM, Role Modeling; SE, Sensory Exploration; SR, Self-Regulation; TEACHER, teacher-focused responsive feeding practice training intervention arm; TEACHER + CLASS, intervention arm including TEACHER plus additional training for classroom nutrition curriculum.

Scores for each section (minimum 0, maximum 10) ranged from low (1.7 for sensory exploration in the TEACHER group) to high (9.5 for rewards and praise in the TEACHER group). The mean baseline score for both TEACHER and TEACHER + CLASS was slightly above midline at 6.1 and 6.5, respectively. There were no changes from baseline to postintervention for the combined 9 programs (data not shown). Differences between baseline and posttraining observations for both groups are presented in **Table 3**. Total MOCC scores improved for the TEACHER, but not for the TEACHER + CLASS, group. There were no significant changes in the role modeling or sensory exploration behaviors. Visual observation of raw data in the viable pairs of baseline–postintervention peer modeling values indicated a significant change for the TEACHER group only, increasing from the lowest possible 0 to the highest possible 10. Although approaching significance, there were no significant changes in supporting self-regulation scores, rewards and praise behaviors, and permissiveness and indulgence behaviors from baseline to postintervention. There were no significant changes in overall feeding styles for the TEACHER group. There were no changes from baseline to postintervention for the TEACHER + CLASS group. See **Figure 1** for section scores.

**TABLE 3:** Baseline and postintervention total Mealtime Observation in Child Care scores and section scores^1^

	TEACHER (*n* = 4)			TEACHER + CLASS (*n* = 5)		
Variable	Baseline	Post	*P* value	Baseline	Post	*P* value
Total Mealtime Observation in Child Care Score	6.1 ± 0.9	7.5 ± 0.3	0.026*	6.5 ± 0.8	6.4 ± 1.0	0.915
Children Serve Themselves Section Score	6.2 ± 1.1	6.4 ± 0.5	0.787	6.4 ± 0.8	6.4 ± 1.2	0.964
Role Modeling Section Score	8.1 ± 1.3	8.5 ± 0.3	0.640	8.2 ± 0.7	8.1 ± 0.5	0.674
Sensory Exploration Section Score	1.7 ± 1.9	4.2 ± 1.7	0.216	3.3 ± 2.4	3.3 ± 2.4	1.000
Peer Modeling Section Score	3.3 ± 5.7‡	10.0 ± 0.0‡	¥	5.4 ± 5.1‡	4.5 ± 2.1	0.801
Self-Regulation Section Score	4.9 ± 1.3	6.0 ± 0.7	0.056	6.8 ± 1.1	5.6 ± 1.2	0.107
Rewards and Praise Section Score	9.5 ± 1.0	7.6 ± 0.6	0.069	8.6 ± 2.2	8.6 ± 1.3	1.000
Permissiveness and Indulgence Section Score	6.3 ± 2.5	10.0 ± 0.0	0.058	5.0 ± 0.0	8.0 ± 2.7	0.070
Overall Feeding Style Section Score	7.5 ± 2.1	9.2 ± 1.7	0.418	7.3 ± 0.9	6.7 ± 2.4	0.578

^1^Values are means ± SDs unless indicated otherwise. Minimum and maximum possible scores for each section and the total score are 0 and 10, respectively. *Significant at *P* ≤ 0.05. ‡*n* = 3. ¥ = limited sample size and no variation precluded statistical analyses and visual evaluation was needed. TEACHER, teacher-focused responsive feeding practice training; TEACHER + CLASS, teacher-focused responsive feeding practice training plus classroom nutrition curriculum.

## Discussion

This study examines the impact of a brief intervention to enhance ECE teachers’ responsive feeding practices in 2 intervention groups, 1 receiving only the teacher-focused responsive feeding practices training and the other receiving the responsive feeding practices training in addition to a training to implement a new classroom nutrition curriculum. The study hypothesis was that after the EAT Family Style intervention training on responsive feeding practice, teacher use of responsive feeding practices, specifically role modeling, use of responsive language to cultivate peer modeling, and support of self-regulation, would increase for both groups. The primary findings were that most responsive feeding practices (total feeding practice score, encouraging peer modeling, encouraging self-regulation, and use of permissiveness and indulgence) increased in the group that received only the teacher-focused responsive feeding practice training but not the group that received the teacher-focused training plus the new classroom curriculum simultaneously. The use of role modeling and encouraging children to serve themselves was unchanged in both groups after the responsive feeding practice intervention. The observation that responsive feeding practices increased for 1 group, but not both groups was contrary to the hypothesis and is an important finding to discuss. In addition, this study demonstrates the feasibility of collaborating with NA tribal partners to implement health interventions in their ECE programs because few interventions have been conducted in this environment (38, 39).

Interestingly, in the group that demonstrated changes, peer role modeling improved whereas teacher role modeling did not. The absence of change in teacher role modeling may be due in part to the high level of role modeling observed at baseline which left less room for improvement. It is unsurprising that the teacher role modeling scores were high at baseline because many of the site managers reported having written policies regarding the use of role modeling, indicating that this feeding behavior was introduced and practiced to some degree before the responsive feeding practices training. Studies have reported peer modeling to be more influential on consumption than is teacher role modeling (20, 40), and that children making negative comments about food can dissuade other children from trying foods (41). During the training planning period of our study, teachers expressed concern about handling food refusal and how to utilize responsive language to encourage peer modeling. The content of the EAT-Family Style intervention was targeted and adapted to meet teacher needs as part of the CBPR process. Approximately 30 min of the training were used to discuss modeling, including a 10-min group discussion on some of the common reactions of children when refusing to try foods and ideas on how to respond. The use of interactive application, such as that used during group discussions, has been shown to improve adult learning (42, 43) and likely improved teachers’ understanding and confidence in using responsive language.

The use of responsive language not only helps encourage peer modeling, but also helps children regulate their intake (11). One study showed that when teachers asked children if they were “full” before removing their plate, the children's intake of fruits and vegetables increased (22). Theories on adult learning have indicated that a change in perspective is necessary for behavior modification to occur (44). During the teacher training, an explanation of self-regulation was given and misconceptions about a child's inability to self-regulate intake, which has previously been reported as a barrier (31), were addressed. In addition, a short video was shown regarding supporting children's self-regulation. Videos, which convey information through visual images and auditory signals, are a favored adult learning tool (45) that can facilitate behavior change (46) and increase knowledge of relevant concepts (47–49). As self-regulation scores increased, replacing teachers’ use of restriction or insistence, we would expect to see an increase in permissive feeding behaviors as we did in this study.

Aligned with the Academy’s benchmarks of Nutrition and Dietetics, teachers have previously reported supporting children's self-regulation in energy intake, and agreed that children should serve themselves and choose their own serving sizes (26, 50). However, institutional-level changes are necessary for teachers to have the needed resources to change serving styles and facilitate children's self-service of food during meals. For instance, appropriate-sized serving dishes would be needed. Of the 9 sites, 8 reported having current policies in place to support family-style meals. The section score representing children serving themselves was slightly above midline scoring for both groups. At baseline, many of the teachers encouraged children to serve all or part of their meals, perhaps limiting room for improvement. Not surprisingly, the children serving themselves score did not change from baseline to postintervention. Previous literature indicates that teachers have expressed barriers to family-style dining including increased messes, food wastage, and staff and resources needed (45). Future research that follows up with the current study of NA ECE programs and the teachers to understand their challenges and provide a follow-up training and resources to address their specific challenges may improve implementation of family-style dining.

Effective interventions include multiple levels in an ecological model (51). However, no previous studies, as far as we know, have examined an intervention addressing how teachers interact with children during mealtime. Previous interventions have taught providers children's health curriculums aiming for classroom integration (39, 52), whereas others have focused on the teachers themselves and addressed their lifestyle habits to better role model for the children (53). The goal of our study was to give teachers the power and motivation to make informed choices about the best way to role model, teach, and communicate with their children. During our study, 1 group of teachers was trained on teacher-focused responsive feeding practices within 2 wk of being trained on the child-focused classroom nutrition curriculum. The other group of teachers was trained only on teacher-focused responsive feeding practices. In light of findings that the TEACHER group experienced the change whereas the TEACHER + CLASS group did not, this may indicate that the 2-wk period did not allow teachers enough time to practice and implement the responsive feeding practices before learning and implementing new classroom materials. Thus, provider priority may have been diverted, resulting in no changes being observed in the TEACHER + CLASS group. Alternatively, it is possible the small sample size was a limiting factor in detecting differences.

Theories on adult learning assume that adult application of learning becomes more immediate and problem-centered (42). Therefore, the sequence of the curriculum must be timed to allow developmental tasks to be completed before moving to the next task. Examining the appropriate amount of time needed for teachers to understand and implement tasks is important to consider when designing interventions. In the present study, the EAT Family Style intervention (26, 31, 50, 54) was adapted to deliver targeted content in 1.5 h to meet the needs expressed by the community. Future training can be conducted based on the original design of the EAT Family Style intervention to evaluate changes in behavior based on the practices included in the full EAT Family Style Intervention. Adults are also assumed to build their knowledge on previous experiences (42). Teachers that have received previous training on a subject may be able to understand and apply the information more easily. However, a change in a teacher’s perspective can promote learning through contemplation (44). For example, a teacher may learn that their assumption that a child does not have the ability to self-regulate intake is inaccurate. If the teacher alters their behavior related to their new understanding and gains personal experience that the child is able to self-regulate intake, their conviction of the need to maintain the transformation will become stronger.

### Strengths and limitations

This is one of the first studies to examine the outcome of a responsive feeding practice intervention with ECE providers. In addition, this study partnered with an NA tribe and their 9 ECE programs to improve the health of children living on the reservation, a population that is at higher risk of obesity. However, it is important to note that these findings may not be representative of all tribally affiliated programs across the state of Oklahoma or the United States. One trained observer collected all observation data for the study, thus eliminating the potential for interrater reliability error. The observer was trained and discussed issues and situations with tool co-developers, which enhanced data quality and integrity and intrarater reliability. Furthermore, using observation data instead of provider self-report enhanced the accuracy of feeding practices.

A strength of the partnership was in scheduling the intervention training at a time established for teacher in-service training to enhance provider participation and attendance. To maintain a strong relationship with the community consistent with CBPR practices and to ensure that participants did not feel they were being individually evaluated, provider demographic characteristics were not collected nor were the names of individual providers observed. This limits the investigators’ ability to verify that all teachers observed were consistent from baseline to postintervention and present at the training. During observation, although individual names could not be recorded, it was noted that some of the teachers in the room were not in attendance at the training and perhaps served other roles at the program, such as administrative or food preparation, and would not have been included in the in-service training, although they were ECE staff.

One limitation was that not all items on the tool were able to be observed, and this novel tool was not validated in NA ECE programs. This limitation made it difficult to compare scores between baseline and postintervention because some data were only observed at 1 time point, and difficult to compare with other studies because the tool is novel. This was accounted for in the tool scoring and those items unable to be scored did not negatively affect the score. Still, understanding whether a behavior occurs or does not occur, rather than only recording that it could not be observed, would be ideal for understanding training effects. Owing to limited resources, feeding behaviors were determined by 2 lunchtime observations which may not have been representative of typical mealtime behaviors. Another potential limitation is that teachers may have altered their behavior in the presence of a researcher in the classroom. The researcher did not interact with the children and had little interaction with the teachers, encouraging them to maintain their usual routine. Teachers altering their behavior may decrease if researchers conduct more observations more frequently, as their presence would become more familiar. Not having control over what other trainings or programs the schools were enrolled in during the course of the semester in which the lunchtime observations were conducted may have introduced confounders to this study. Finally, this study had a small sample size with a total of 9 sites (5 TEACHER + CLASS intervention programs in 2 communities and 4 TEACHER intervention programs in 2 other communities).

### Future directions and practical applications

Based on the findings counter to our original hypothesis and an in-depth review of adult learning theoretical approaches, we conclude that the healthful feeding practices training did have positive impact on aspects of feeding practices, such as cultivating peer modeling and supporting self-regulation, in the TEACHER group, but not in the TEACHER + CLASS group. Although the sample size was small, an important implication for practice would be to ensure that interventions that include teacher training must account for adequate time between content areas to incorporate new concepts into classroom and personal application. Although this approach may take longer, it may ensure that the content is internalized by teachers, resulting in a positive impact on classroom quality and child health. Future studies should explore the relation, in terms of intervention timing, between healthy feeding practices training and classroom curriculum. Few research tools have been developed and validated in NA populations and this too is an area for future research. Further, this project demonstrated the feasibility of collaborative partnership with NA communities to enhance the health of young NA children. This study can serve as a platform upon which future collaborative opportunities can be built.

Given the aforementioned limitations, some suggestions for future research are salient regarding the frequency of data observation and rigor in collecting intervention attendance and tracking individual teachers in the classrooms of observation. Including observed teachers’ names will allow for more sophisticated intent-to-treat analyses to determine true intervention impact. Although these data were not recorded in this project at the request of the community, it will be important for future projects working with ECE, tribal and otherwise, to advocate for the ability to record which teachers are present at trainings and observations to determine the impact of the training intervention. To address community concerns about criticism and privacy, care should be taken to communicate that the purpose is not to evaluate individual teachers but to ensure that teachers being observed were actually exposed to the training and thus evaluating the training effectiveness. Future studies should aim to work with more communities to provide greater statistical power. Including more observation time points would also provide a more accurate understanding of typical mealtime interactions and increase the likelihood of being able to produce a score for all items on the tool both pre- and posttraining. This would potentially address the social desirability bias if teachers were modifying behaviors for a single day of observation.

## Conclusions

Teachers’ feeding behaviors shape children's food intake and eating behaviors (11). This study was one of the first to explore the effect of teacher training on responsive feeding behaviors. Surprisingly, results indicate improvement in the teacher-focused group, but not in the teacher and classroom group who, within 2 wk, were trained on and concurrently began administering an intensive 15-wk classroom nutrition curriculum. Although the sample size in this study was not large, it is clearly important to consider this when designing, planning, and implementing trainings for future interventions. One assumption of the adult learning theory is that adults prefer problem-centered information that can be applied to more immediate needs (42). Whereas the TEACHER group had time to apply and adopt the information from the training without other training demands, the TEACHER + CLASS group may have been overwhelmed with the many tasks related to implementation of the broader class nutrition curriculum and deprioritized the responsive feeding practice training. These findings may indicate that more time is needed to implement one task before another is added. Recognizing how to effectively help teachers understand and implement healthy feeding practices will facilitate children's ability to develop healthy eating patterns and make healthy choices, which could be protective against developing chronic diseases such as cancer and diabetes later in the life course.

## Notes

Supported by National Institute on Minority Health and Health Disparities grant R01MD011266 (to VBBJ).

Author disclosures: KS, SBS, DAD, CL, MBW, LAH, and VBBJ, no conflicts of interest.

The funding agency did not participate in the study design, data collection, analysis, decision to publish, or preparation of the manuscript.

The data used in this study are not deposited in a general repository. Opportunities to collaborate should be directed to the corresponding author.

This article appears as part of the supplement “Proceedings of the Third Annual Conference on Native American Nutrition,” sponsored by the Shakopee Mdewakanton Sioux Community's Seeds of Native Health campaign through a gift to the University of Minnesota. The guest editors of the supplement, Treena Delormier and Mindy S Kurzer, have no conflicts of interest. The opinions expressed in this publication are those of the authors and are not attributable to the sponsors or the publisher, Editor, or Editorial Board of *Current Developments in Nutrition*. Publication costs for this supplement were defrayed in part by the payment of page charges.

Abbreviations used: CBPR, community-based participatory research; EAT Family Style intervention, Ecological Approach To Family Style Dining Intervention; ECE, early care and education; MOCC, Mealtime Observation in Child Care; NA, Native American; TEACHER, teacher-focused responsive feeding practice training intervention arm; TEACHER + CLASS, intervention arm including TEACHER plus additional training for classroom nutrition curriculum.
